# Transitions in Technology-Mediated Cardiac Rehabilitation and Self-management: Qualitative Study Using the Theoretical Domains Framework

**DOI:** 10.2196/30428

**Published:** 2021-10-14

**Authors:** Shreya Tadas, Claudette Pretorius, Emma J Foster, Trish Gorely, Stephen J Leslie, David Coyle

**Affiliations:** 1 School of Computer Science, University College Dublin Dublin Ireland; 2 Cardiac Unit, NHS Highland Inverness United Kingdom; 3 Department of Nursing and Midwifery, University of the Highlands and Islands Inverness United Kingdom; 4 School of Health, Social Care and Life Sciences, University of the Highlands and Islands Inverness United Kingdom

**Keywords:** cardiac rehabilitation, self-management, self-care, behavioral change, Theoretical Domains Framework, qualitative methods, mobile phone

## Abstract

**Background:**

An acute cardiac incident is a life-changing event that often necessitates surgery. Although surgery has high success rates, rehabilitation, behavioral changes, and self-care are critical to long-term health. Recent systematic reviews have highlighted the potential of technology in this area; however, significant shortcomings have also been identified, particularly with regard to patient experience.

**Objective:**

This study aims to improve future systems and to explore the experiences of cardiac patients during key phases after hospitalization: recuperation, initial rehabilitation, and long-term self-management. The key objective is to provide a holistic understanding of behavioral factors that impact people across these phases, understand how experiences evolve over time, and provide user-centered recommendations to improve the design of cardiac rehabilitation and self-management technologies.

**Methods:**

Semistructured interviews were conducted with people who attended rehabilitation programs following hospitalization for acute cardiac events. Interviews were developed and data were analyzed via the Theoretical Domains Framework, a pragmatic framework that synthesizes prior theories of behavioral change.

**Results:**

Three phases that arise posthospitalization were examined, namely, recuperation, rehabilitation, and long-term self-management. Through these phases, we describe the impact of key factors and important changes that occur in patients’ experiences over time, including the desire for and redefinition of normal life, the need for different types of formal and informal knowledge, the benefits of safe zoning and connectedness, and the need to recognize capability. The use of the Theoretical Domains Framework allows us to show how factors that influence behavior evolve over time and to identify potential sources of tension.

**Conclusions:**

This study provides empirically grounded recommendations for the design of technology-mediated cardiac rehabilitation and self-management systems. Key recommendations include the use of technology to support a normal life, leveraging social influences to extend participants’ sense of normality, the use of technology to provide a safe zone, the need to support both emotional and physical well-being, and a focus on recognizing capability and providing recommendations that are positive and reinforce this capability.

## Introduction

### Background

**Figure 1:**
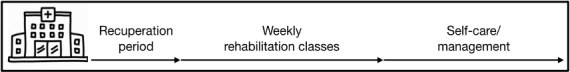
Participants’ journey after their cardiac event.

Cardiovascular disease (CVD) is a leading cause of morbidity and mortality worldwide, with an estimated 17.9 million deaths each year [1]. CVDs are a group of disorders of the heart and blood vessels, usually associated with a buildup of fatty deposits inside the arteries that occur when the flow of oxygen-rich blood to the heart is blocked, leading to increased strain on the heart [2]. Four out of five cardiac-related deaths are due to acute events, such as heart attacks and strokes. One-third of these deaths occur prematurely in people under 70 years of age [1]. Fortunately, the success rate of modern cardiac surgery and nonsurgical interventions, such as percutaneous coronary intervention (stent insertion), is high. As a result, an increasing number of people live with CVD as a long-term chronic condition. Following acute events, which are often sudden, ongoing treatment for CVD involves lifestyle changes and medicines. Cardiac rehabilitation is considered a vital part of long-term recovery and a key component of patient management. It may include clinical assessment, medication review, risk factor modification, psychological support, and supervised exercise [3]. After a person is hospitalized and after discharge, they go through a recuperation period (Figure 1). Many patients attend rehabilitation programs. Individualized cardiac rehabilitation programs are usually for a duration of 6-8 weeks and comprise a mix of monitored exercises and educational sessions. Following these initial stages, people must continue self-management. According to Barlow et al [4], “self-management refers to the individual’s ability to manage the symptoms, treatment, physical and psychological consequences and lifestyle changes.” Many people face emotional and physical challenges during the transition from hospitalization to self-management [5]. For successful and long-term self-management, behavior and behavioral change play a central role [6]. Evidence suggests that rehabilitation programs can play a vital role in the transition from hospitalization to self-care [7]. Despite the proven benefits, uptake and adherence in traditional face-to-face programs are often low due to barriers such as lack of awareness, transport, motivation, cost, and poor social support [8].

Recent systematic reviews have highlighted the potential of digital health interventions to support rehabilitation and subsequent self-management of cardiovascular conditions [9-11]. However, significant shortcomings were identified. Evidence suggests that tightly supervised intervention programs are most successful and that self-directed management is less successful due to problems with engagement and adherence. Piette et al [10] highlighted the need for future interventions to incorporate advances in behavioral theories and artificial intelligence in order to be more effective and adaptive to the changing needs of patients. Despite recent calls for technology that supports personalization and focuses on user needs, Tadas et al [11] found that, with notable exceptions, prior research in the cardiovascular domain has made limited use of user-centered approaches. This is consistent with the findings of Siegers et al [12], who also reported that most developers of digital interventions for cardiac self-management did not engage with the direct experiences of patients, such as those who have attended rehabilitation programs. Prior studies have also tended to focus on specific aspects of self-management, such as physical activity [13] and medication management [9,14]. They do not provide a holistic understanding of the behavioral factors that impact people throughout recuperation, rehabilitation, and self-management.

This paper builds on recent research on posthospital transitions [5,15] and on rehabilitation and self-management in chronic conditions [16,17]. It responds directly to calls for research in the cardiovascular domain to engage more deeply with both behavioral change theories and with patient experience. The contributions of this paper include a comprehensive assessment of people’s experiences of recuperation, rehabilitation, and self-management, their attitude toward technology, and the ways in which it could better support rehabilitation and self-care. The analysis is framed via the Theoretical Domains Framework (TDF), an integrated theoretical framework synthesized from 33 prior theories of behavioral change. The key strength of the TDF is that it provides a rigorous and comprehensive framework to identify factors that impact behavior and behavioral change. Our analysis was grounded in a semistructured interview study with 19 participants who were hospitalized following an acute cardiac incident and subsequently attended a cardiac rehabilitation program.

Our research questions include:

1. What were the key experiences of patients after cardiac surgery and how did these experiences support or hinder rehabilitation and ongoing self-management?
2. How did the experiences of patients change over different phases of recuperation, rehabilitation, and self-management?
3. What strategies can be applied in design to better support technology-mediated cardiac rehabilitation and self-management?

### Related Work

#### Overview

The work presented in this paper builds on existing research in a number of key areas, including literature on posthospitalization transitions and support, rehabilitation, and self-management in chronic conditions, and theories and frameworks for behavioral change. We begin with an overview of key recent work specific to cardiovascular care and then consider related work outside of the cardiovascular domain. In addition to research in the health domain and reflecting the emphasis in the paper on understanding user experience, we consider relevant literature in the field of human-computer interaction (HCI).

#### Technology in Cardiovascular Care

Technology-mediated cardiovascular rehabilitation and self-management has generally been provided through mobile apps, web apps, sensors, or an integration of these [11]. These systems aim to increase adherence, motivation, and engagement through different means, including gamification, guidance, and education about the condition, reminders, and data tracking through sensors. Most of these studies have focused on interventions to increase physical activity and exercise. Some aim to provide a medium for better communication and data sharing between patients and care providers, nurses, or health professionals. A small number facilitate remote cardiac rehabilitation. A recent systematic review [9] concluded that mobile apps in particular offer an important opportunity to improve access to secondary prevention for cardiac patients, but also concluded that this potential has not been achieved to date. The authors stress the need for personalized and user-friendly apps that can cater to the needs of individual patients from different age groups. A systematic review of mobile health apps for CVD, including commercially available apps, by Athilingam et al [18] identified a trend toward cost-effectiveness and potential solutions for symptom monitoring and promoting patient engagement in their own homes. However, evidence of impact on heart failure–related outcomes is inconclusive. The review also found that most apps focused on monitoring patients’ symptoms and activity but provided limited feedback on unusual or irregular events. Andersen et al [19] emphasized the importance of aligning concerns of patients and clinicians and proposed three key concepts to consider while designing eHealth systems: the system should be meaningful and actionable to both clinicians and patients and feasible within the organizational and social context. Another study by Andersen et al [20] demonstrated the use of user-centered design methods for reintroducing patients as active diagnostic agents to design a collaborative digital tool for monitoring heart patients after hospitalization. This study emphasizes the importance of increasing patient participation in the design of eHealth systems and telemonitoring practices. Similar studies on posthospital transitions in chronic patients describe how discharged patients are often unprepared to self-manage their condition at home [5]. Being discharged from hospital meant a transition from a safe environment at the hospital to an unknown environment at home [15,21]. The transition of people with cardiac conditions from hospital after surgery to their homes is equally likely to create challenges and design opportunities, which this paper seeks to address; there is a need for more participatory and iterative approaches to design patient-centered eHealth systems [19,22]. A qualitative systematic review by Tadas et al [11] identified the limited use of user-centered design methods and theoretical models to guide the design of technology for cardiovascular care.

Digital apps for cardiac-specific rehabilitation and self-management are focused on physical activity monitoring [23], virtual rehabilitation programs [24], medication management [14], and heart rate and blood pressure monitoring [25-27]. Although recent digital apps show effective results, self-management and rehabilitation using digital apps generally show a gradual decline in use over time due to resistance to behavioral change and lack of motivation [14]. Investigations by Maitland et al [28] on the role of self-monitoring found an overall reluctance toward unnecessary self-monitoring and suggested that technology should focus on self-awareness and self-determination. Overall, there is a need for more research directly examining the experiences of people after cardiac events in relation to digital tools to support cardiac rehabilitation and self-management.

The HCI community, which promotes and practices user-centered design methods, has relatively less research on technologies for cardiac conditions, an observation also noted by Nunes et al [17] in their comprehensive review of HCI research on self-care technologies. Of the 30 studies included in their review, only 3 addressed cardiac conditions. Diabetes was found to be the most common condition addressed by the HCI research on self-care technologies. Examples of self-care technology used for diabetes management include the AssistingInsulin smartphone app by Preuveneers et al [29], which recommends insulin dosages based on predictions of the user’s activity, and exploration of contextual frames by Raj et al [30] that demonstrates the relationship between context and behavior and the importance of context-aware apps for self-management. Furthermore, recent research shows an increasing demand for self-management technology that supports people’s mundane activities and informal ways of exercise [31]. Significant research also exists in the space of self-management technologies aimed at addressing chronic disease management in older adults [16,32,33]. For example, the study on managing multimorbidity in older adults by Doyle et al [16], suggests the need for self-management apps to primarily focus on information support and teaching how to self-manage. There is also a growing body of work targeted at supporting chronic obstructive pulmonary disease therapy and training at home with the use of sensors, smartphones, television, and webcams [34,35]. Research in this area demonstrates the increasing accuracy of smartphone-based training apps and their acceptance. Existing research on other chronic conditions has clear relevance for the cardiovascular domain. However, to be most effective, we require a detailed understanding of the specific requirements of the people experiencing CVD.

#### Theoretical Domains Framework

Behavioral change theories and methodologies have been widely applied to guide the design of technical systems and evaluation strategies [36-38]. A systematic review exploring the potential of web-based self-management programs found that systems that incorporated behavioral change techniques were more effective than those that did not, and that web-based systems were more effective than no intervention [39]. There are many theoretical models of behavior, including the Health Belief Model [40], the theory of reasoned action [41], the theory of planned behavior [41], and the social cognitive theory [42]. Although a large number of theoretical models present opportunities, they also create challenges. Many theories either include a small number of constructs or share common or overlapping constructs, such as intention, social norms, beliefs, or control or self-efficacy. Therefore, in some cases, it is difficult to decipher which the most appropriate factors to target are in behavioral change interventions. In other cases, it is also possible that the key determinants of the target behavior are not represented. TDF [10,43] was developed in response to these challenges, in an effort to assimilate overlapping constructs in a pragmatic framework and to improve researchers’ access to and application of psychological theory.

TDF is an integrated theoretical framework composed of domains synthesized from 33 prior theories and 128 theoretical constructs relevant to behavioral change [43]. It was developed in collaboration with behavioral scientists and implementation researchers to provide a comprehensive and holistic approach to identify determinants of behavior and potential targets for behavioral change. The TDF contains 14 domains covering 84 constructs, examples of which include *environmental context and resources*, *emotion*, *goals and intentions*, *beliefs about capabilities*, *knowledge and skills*, and *social influences*. A complete listing of the domains and the constructs related to each is available in Lou Atkins et al [43]. TDF was initially developed to identify influences on health professional behavior, but has been extended to many areas in which changing behavior is important, including changing patient behavior [43]. It supports the assessment of problems and identification of potential solutions by providing a lens to view the cognitive, affective, social, and environmental influences on behavior. As a pragmatic framework, it signals opportunities and methods for intervention by first identifying key domains and constructs and subsequently providing a guide to relevant explanations of current behaviors [10].

TDF has been widely used in health research, particularly for qualitative approaches [44]. Examples of qualitative studies include using TDF to formulate interview questionnaires to address target behavior [45,46], to analyze interview responses to identify barriers and facilitators in implementing interventions for families of people with schizophrenia [47], and increasing physical activity in stroke survivors [48]. In applying TDF, we aimed to identify key determinants of behavior in cardiac rehabilitation and self-management at the individual level. We also aimed to explore the key barriers and facilitators to implementing technology-mediated cardiac rehabilitation and self-management solutions. In this paper, we use TDF in the following ways: (1) as a basis for the interview questionnaire to explore individual motivation and capability factors while also covering the physical and environmental influences; (2) to identify the relevant domains that are most likely to influence technology-mediated cardiac rehabilitation and self-management and associated behaviors; and (3) to identify the key points during recuperation, rehabilitation, and self-management journey when different domains exert a strong influence on peoples’ experiences and behaviors. The key advantage of TDF is that it provides a pragmatic, yet rigorous, and holistic framework to address these issues.

## Methods

### Overview

We conducted semistructured interviews with people who had been hospitalized due to a cardiac event and subsequently attended supervised rehabilitation programs. Interviews were framed using TDF and explored participants’ journeys and experiences after hospitalization, their cardiac rehabilitation experiences, and their attitudes toward technology. Thereafter, as supported by the TDF guidelines, we performed an inductive analysis of the interview responses following the Braun and Clarke thematic approach [49].

### Recruitment

This study was conducted in collaboration with the cardiac unit at Raigmore Hospital, a National Health Service (NHS) Trust in the United Kingdom. A total of 19 participants (11 women) were recruited. All participants had had either a cardiac incident or a cardiac disease in the past. All participants were offered a postsurgery cardiac rehabilitation program at the Raigmore Hospital [50]. The program consisted of a mix of education sessions and monitored exercises. To represent a range of views, we recruited patients who had attended some, but not all, rehabilitation classes and others who had attended all classes (Multimedia Appendix 1). The exclusion criteria were adolescents and people with severe cognitive impairment or terminal illness, as it was outside the scope of this study. Participants’ ages ranged between 50 and 86 years, mean 70 (SD 9).

### Procedure

This study was approved by the Health Research Authority, NHS Research Scotland, and the Human Research Ethics Committee, University College Dublin. A total of 52 patients were sent interview requests over post. Nineteen patients agreed to participate in the study. The interviews were conducted separately over telephone calls and audio recorded. Each interview took approximately 45 minutes.

The interview questions were based on TDF and inquired about patients’ experiences after cardiac surgery, focusing on domains of TDF related to knowledge and skills, individual goals and intentions, social and environmental influences, and emotional influence [43]. All TDF domains were examined, and only those relevant to the aims of this study were considered. This is consistent with the guidelines for the use of TDF. Questions about knowledge and skills inquired about their help seeking, new skills or techniques considered after cardiac events, sources of information, and awareness of their cardiac condition. This included, but was not limited to, awareness of support resources such as mainstream self-care technologies and rehabilitation programs. Individual goals and intentions questions were about their experience of the rehabilitation program and its barriers and facilitators, posthospitalization life goals and changes, and progress tracking. Questions about social and environmental factors probed environmental and social sources of influence and motivation, including the role of health experts and technology on self-management postcardiac events. Questions about emotional influences focused on emotional reactions and feelings of after cardiac events.

The interview questions were structured according to each phase the participant went through after hospitalization (Figure 1). The interview protocol was designed in collaboration with all the authors and is available in Multimedia Appendix 2. The interviews were conducted by the first author (ST), who has a background in HCI and is an experienced qualitative researcher. The semistructured interview started with questions about the participants’ first cardiac incident, including hospitalization, initial awareness about the cardiac condition, and support resources. This was followed by questions related to the rehabilitation program experience, and then the self-management experience.

### Analysis

Audio recordings of the interviews were transcribed verbatim. The transcripts were analyzed using the NVivo 12 (QSR International) software and inductively coded using a thematic approach following the Braun and Clarke methodology [49]. A codebook was created through an iterative process of coding and clean coding. This was performed by dividing 30% of the total interviews between the two authors. Coding was performed using an inductive approach. Conflicts were discussed and resolved through discussions with a third researcher. After reaching a consensus on the codebook, three randomly selected interviews from the entire data set were coded. The Cohen κ coefficient was computed to assess the interrater reliability at this point, with a score across all codes of 0.53. Further discussion and refinement of codes took place to clarify and agree with the final set of codes. On the basis of this final codebook, the remaining transcripts were coded by the first author (ST). Following coding, themes were identified and again reviewed and defined through an iterative process of independent and group analysis involving all 3 authors.

## Results

### Overview

An analysis of interviews with participants about their posthospitalization experiences identified a number of key themes. In Table 1, these themes are categorized into the three key phases the patients went through after hospitalization, namely, recuperation, rehabilitation, and self-management. As shown in Table 1, the findings are also classified in the context of the TDF domains. It is important to note that there is some overlap in the themes identified in Table 1, with issues present in more than 1 phase. Our analysis deliberately placed emphasis on identifying the themes in each phase. This has resulted in more overall themes than might typically be the case in thematic analysis. However, structuring our findings in this way has the benefit of allowing us to identify the point when particular experiences first emerged, and when they were felt most strongly. In the Discussion section, we will reflect on how specific needs (eg, a desire for normality) change over time and the implications these changes have on the design of technology.

**Table 1:** Mapping of posthospitalization transition phases, relevant Theoretical Domains Framework (TDF) domains, and themes from findings.

Transition and TDF domains		Themes	Codes
**Recuperation phase**			
	Goals	A desire for normality	Feeling better after cardiac event Rebuilding strength Desire for a normal life
	Knowledge	Sources of information and role of official or expert resources	Initial help seeking Need for information Contact with health care professionals Resources recommended by experts
	Emotion	Shock and gratitude	Gratitude or appreciation Emotional response or reaction
**Rehabilitation classes phase**			
	Emotion Optimism	Mindset and emotion	Stress or anxiety and relaxation Positivity or negativity Fear
	Environmental context and resources	Rehabilitation classes provide a safe space	Preference for local or in-person rehab Rehabilitation classes as a training place Classes as a safe zone Tailoring Barriers to local attendance
	Social influences	Rehabilitation classes provide a social space	Rehabilitation classes as a social place
**Self-management phase**			
	Environmental context and resources Social influences	The importance of family and social support	Environmental or contextual support Social support and types of social support Self-reliance
	Behavioral regulation	Monitoring	Bodily awareness Monitoring Motivation or demotivation
	Beliefs about capability	Capability	Emphasize what can be done Physical activity found in daily activity

### Recuperation Phase

Recuperation phase is the period immediately after discharge from hospital following cardiac surgery.

#### Desire for Normality

The desire for a normal life (defined as the life patients had before cardiac surgery) was identified across each of the three phases described in our results. However, this desire first emerged and was expressed most strongly during the recuperation phase. While some patients experienced significant physical and mental effects, other patients described feeling better and healthier after surgery. Some went so far as to say procedures such as the insertion of stents had *fixed them* (ie, cured the cardiac problem) and given them confidence to return to normal life:

Once the stents had been fitted, the pain had disappeared, and I felt that the care that I was getting in hospital gave me the confidence to go ahead.

P19

I don’t have a condition as far as I am concerned. I had the operation repaired and I’ve never looked back.

P8

Others spoke positively about their posthospitalization recuperation but described a more step-by-step, gradual process of rebuilding strength. Every day, they would push themselves to do more, but in small increments:

Right enough, the next day I went out, I got a bit further. The day after that, a bit further. That was fine. So, I didn’t actually have any low points. I didn’t regress much at all. It was a fairly gradual and continuous improvement.

P13

Overall, participants expressed a strong desire to lead a *normal life* after the cardiac event, without the need to be reminded of their condition. Although hospitals provide a lot of information during discharge on potential risks and the importance of aftercare, many were more interested in knowing how and when they could return to their normal way of living:

They had a lot of information on the aftercare definitely, what we should do, but I was more interested in would I return to my normal things ‘cause I’m a physical person. I’m a walker and I’m always very active and they encouraged me to carry on just like that.

P3

I think we all change a wee bit but the whole point is, is not to make a fuss about it, you have to try and get back in your routine again with your family as much as possible and keep it as normal as possible.

P17

This desire was also expressed with regard to relationships. People wanted to be treated as normal by their friends and family, that is, not being overcared for. They wanted *to get on their feet* and participate in family life in the same way they had normally done before the incident:

Just treating you I suppose how you were before the incident, if you know what I mean. You’re not any different. Maybe my family is just like that. Once I was up on my feet, that was it. Mum’s back, sort of thing. I got away with making the Christmas dinner the first year ‘cause I was away at the hospital, but I was back to it the next year. That did help because it makes things seem normal. I’ve had this incident and I can just go on with the rest of the life. So that helped me in that way.

P15

Viewed through TDF, returning to normal life can be seen as a goal of our participants. It is likely to have a strong influence on the participants’ behavior. The fact that this goal is strongly linked to participants’ sense of role and identity (eg, the family role) is likely to act as further reinforcement. However, the goal of returning to their life before surgery creates a potential tension, as it may come into conflict with the lifestyle change goals recommended for rehabilitation and long-term health management. Resolving this tension is therefore important for technology designs in this space.

#### Sources of Information and Role of Official or Expert Resources

The patients stressed the need for information about their condition. Increasing awareness and information is important for building confidence, “Having a heart attack was quite a shock to me and as I said I read as much as I could about it” [P18].

There is a need for reliable information and a need to help people retain this information. Those who had a family history or earlier awareness of cardiac symptoms were better prepared to handle the repercussions. When asked about how they sought information initially, the most common response was from the internet and booklets given by their hospital. However, patients also expressed concerns about the credibility and possibility of harm in seeking information on the internet:

If I had a problem or if I wanted to find out anything about health I will look it up on the computer.

P10

Googling too much messes with the head - panic due to sharp info content.

P4

In the initial stages of recuperation, the resources recommended by experts were highly valued, as patients trusted these resources. Participants were strongly of the view that there was a need for access to and contact with experts and health professionals after discharge. Any type of contact with health professionals was found to be reassuring during the transition from hospital to self-care and recovery. Talks from experts in rehabilitation programs were considered valuable. However, this contact was sometimes restrained because of time restrictions on health professionals. However, it is also due to concerns on the part of patients that they might burden health professionals:

Maybe just more contact or freer to contact the cardiac advice line because, me personally, you tend not to want to be contacting them unnecessarily but sometimes just after in the first two or three months...It’s just that you feel that you weren’t encouraged to do it. No one said, “Just contact us if you’re concerned about anything.”

P3

Participants’ desire for information is consistent with the TDF *Knowledge* domain. During the recuperation phase, participants had a need for general knowledge about cardiac conditions and rehabilitation procedures. They placed a strong emphasis on official knowledge sources. As will be seen in later sections, the types of knowledge participants prioritized evolved during subsequent phases, with a greater emphasis on detailed personalized understanding and informal information sources.

#### Shock and Gratitude

Acute cardiac events are typically sudden, and unsurprisingly, trigger strong emotional responses. Some participants were physically fit, had no other earlier health issues, no symptoms, and no one in their family had had heart problems earlier. However, suddenly, they experienced a life-threatening event, were hospitalized, and underwent surgery. This was a significant shock. One participant described being so surprised that it took him a few months to come to terms with the fact that he had had a heart attack. Recovering from such incidents requires both emotional and physical healing,

It was a huge shock to have a heart attack, a real shock to the system, and it just shows you how vulnerable we are and I think that in itself was an incentive.

P18

Following this initial shock, many patients described a newfound appreciation of life and did not want to take their health for granted. They also expressed immense gratitude to and appreciation of health care providers:

I was aware that this is real, what happened to me, and you know, I used to think I was invincible. Well, I never really thought I was anything other than fit and nothing would go wrong, but now I’m aware, much more aware, that something could go wrong, and I’m very grateful for what they did to me.

P16

TDF emphasizes the important role that emotion plays in driving behavior. Participants’ sense of shock clearly shows how the emotions experienced have the potential to drive emotional and physical tension. Interestingly, while shock delayed some patients’ ability to move forward, in others, it helped raise awareness and acted as an incentive. In contrast, gratitude always triggered strongly positive responses during the recuperation phase.

### Rehabilitation Phase

All participants were offered a cardiac rehabilitation program after surgery at a hospital in the NHS Trust [50]. This section discusses the participants’ experiences of rehabilitation classes and this phase more broadly.

#### Mindset and Emotions

The patients’ emotional responses developed and evolved during the rehabilitation phase. Although cardiac events brought out both positive and negative emotions, many described how their mindset or outlook played a major role in recovery and rehabilitation. Participants pointed out that their confidence, determination, and acceptance of their condition helped to reduce the impact of the event on their life:

I’m generally quite a positive person and reasonably confident. I think not unnaturally confident, but if I understand a situation and I know about it and I know what to expect, then I’m fine with it.

P13

Participants realized the importance of reducing stress or anxiety and noted the benefits of relaxation exercises, which were introduced in the rehabilitation classes and were new to many, “I really liked the relaxation type of stuff, I had never done that in my life, never knew anything about that” [P13].

On the contrary, some participants emphasized that a lack of attention to mental health support, after discharge from hospital and in the rehabilitation program had an impact on their recovery. One patient was moved to look for private psychological support outside the public NHS system,

Half of the problem’s with my head to be quite honest with you and if anything I feel that you get let down a wee bit on the recovery part or the mental side of the trauma and I don’t feel there’s enough done in cardio rehab.

P4

Fear was a common emotion during rehabilitation. Some, for example, were apprehensive about pushing themselves to perform exercises as they were constantly afraid of harming themselves. Others expressed a general concern about an uncertain future. Participants felt that this buildup of fear in their minds hindered their progressive recovery and potential for self-management:

I didn’t sleep very well. In fact, I slept in a chair most of the time. It was just apprehension, I suppose, wondering if your life was going – I just thought it was going to drastically change and I wasn’t going to be able to do anything, if you know what I mean. I got over that, but it was always at the back of my mind how much will I be able to do because I didn’t want to be having to just sit about all the time, but that wore off the better that I got. I did pick up quite quickly.

P15

The TDF domain *Emotion* includes the constructs *fear*, *anxiety*, *positive/negative affect*, and *stress*. Helping people address these emotions is clearly an important priority in enabling effective rehabilitation and self-management, but one that may be overlooked in some traditional rehabilitation programs. This emphasizes the importance of supporting both physical and mental health during rehabilitation. Technologies that can provide emotional and mental support, along with reinforcement of a positive mindset and self-reliance, have significant potential in this space.

#### Rehabilitation Classes Provide a Safe Space

Although participants identified barriers, they generally expressed a strong preference for local and in-person rehabilitation. Common barriers reported included transportation, distance, schedule delays, low attendance, limited expert availability, and logistic difficulties. Although the preference for in-person rehabilitation is perhaps unsurprising given the participants recruited, the reasons behind this preference point to important factors for technology design.

Rehabilitation classes provided support for training, giving people the opportunity to gain information and practice physical exercises that they could continue during self-management. They liked the personal interaction with health professionals as it gave them confidence and reassurance that they were doing things properly and progressing. Critically, rehabilitation classes provided a controlled environment—a *safe zone*—while exercising and people felt that they could push themselves without the risk of overburdening their body. This safe zoning was important in helping participants overcome emotions such as fear:

I benefitted greatly from the program – the exercise program. Principally because it was monitored because if I get breathless now doing things, I don’t want to push it because I don’t know how serious that would be, but in the classes when I got nearly breathless, the physio really checked carefully and I felt perfectly relaxed. I knew that nothing untoward would happen while I was in their care.

P9

The patients found that the tailored support focusing on individual needs was encouraging. The rehabilitation program was appreciated for treating every patient individually and helping set appropriate individual goals and where everybody felt they were achieving something. This encouraged them to continue their progress. However, some patients found the rehabilitation classes a bit slow and pointed out that the official self-management information resources received from the hospital were generic. Patients wanted the rate of exercise, type of exercise, and information they received to be determined by their particular needs and how they progressed individually,

My feeling is slightly that each person’s recovery is very individual and not everybody would want to read through the British Heart Foundation.

P1

Importantly, rehabilitation classes also provided a structured approach, compartmentalized physical activity, and monitored to set time, separated from regular day-to-day activities. This was key for some participants, as it supported a sense of normality outside of classes, by allowing for time-bound engagement in physical activity and reserving a set time and place to completely focus on recovery.

TDF emphasizes the behavioral impact of the environmental context and resources. Our findings show that individual and tailored support, safe zoning, and structure or compartmentalization are important elements in the environment provided by rehabilitation classes. Therefore, designs that leverage or recreate these environmental factors have significant potential.

#### Rehabilitation Classes Provide a Social Space

Together with the environmental benefits, rehabilitation classes were also a social place. This provided several clear benefits, consistent with the TDF social influence domain. In particular, it provided a sense of community and gave people the opportunity to talk to others in similar positions,

I think when you are face-to-face with a group of people who are recovering, the same way as you are, I think you encourage each other and I think also the information that you receive collectively adds force to the information that you are given.

P18

In contrast to formal information provided by health professionals during the recuperation phase, information at this point also came in the form of shared experiences. Although this information is less formal, it is also more personal and has collective power. Patients discussed their direct experiences of dealing with various aspects of the recovery process and reassured each other:

One other big advantage was being able to talk to other people who were in a similar position. That was really useful, and I think we could reassure each other, and we could talk to each other about how we dealt with various aspects of the recovery process. That was a very valuable part of it.

P13

A contrast was observed in the case of normality. In the recuperation phase, normality was associated with life before cardiac surgery. The social aspect of rehabilitation classes had the potential to help participants normalize their new experiences, which in turn helped them adjust to a changing life after hospitalization,

The classes were good, mainly the fact that we were talking to people who had gone through the same problem, and come out the other end, and we were getting the feedback from them, making us feel, well, they’ve been through it, they’re looking well, so maybe we can do the same.

P11

Finally, the social nature of the rehabilitation classes was a clear source of motivation. Many participants had experienced technology as *solitary* and not something that was shared with other people. Many were reluctant to replace human contact with technology. *Human touch* was considered very important, whereas technology was considered optional or supplementary,

The motivation is to meet with people and you all join in and that’s the motivation I think and you would find time to go to a class, whereas if you were busy during the day doing other things, you sort of put it off and maybe the grandchildren will come and you want to spend time with them and you think I will do that later, the motivation isn’t there.

P19

### Self-management Phase

Following the recuperation and rehabilitation class phase, participants moved to the self-management phase, requiring them to take greater responsibility to manage their own condition, without regular professional support.

#### Importance of Family and Social Support

Social influence was again a key factor in the self-management phase, but here the focus shifted toward preestablished and longer-term relationships. Family was a key enforcer in every phase, but became particularly important in the self-management phase, with close partners particularly important. Family or partners influenced patients’ physical state by accompanying them for fitness activities or caring for their diet. They influenced their mental state by encouraging and caring, or just being normal:

My wife is very encouraging of me to do healthy things. She leaves it to me, but she’s very positive about it, very helpful. She doesn’t badger me at all, but she encourages me. I think that’s important. If there is someone close to you who cheers your goals and wants you to do well in those goals. I think that makes a huge difference.

P13

Other social support included friends, common interest groups, or web-based support groups. Web-based support groups, although not described favorably by many participants, enabled continuity of communication and mutual support for people who may be living in remote areas or are unable to get together with others:

Interacting with people is much more important because it’s social. It prevents depression. I could quite see how you come home from hospital, you’re living on your own, I’m frightened.

P2

Participants also described the influence of environmental or contextual factors, such as the home, workplace, and surroundings on recovery and self-management. Stress at home and workplace causes anxiety, which could have detrimental effects. Most patients found scenic surroundings and nature walks beneficial:

I’m very lucky. We live in the country, we own our own house, I have a most amazing view from where I’m sitting talking to you just now, and I don’t have pressures that a lot of people will have.

P2

Although many participants valued social support, some patients preferred to be self-reliant, not liking to be told what to do, and wanting to be in control of their lives. Some did not want to be a burden on their family and would not bother their general practitioners unless absolutely necessary. However, 2 patients stressed that they did not need any type of help or support, as they considered themselves to be self-sufficient:

I’m fortunate that I’ve not got people around at all to assist me or help me in any way and that I maintain is a great, because I strive to do these things.

P9

I’ve lived on my own for most of my life and I’m very sort of self-sufficient I suppose, in a way.

P7

In the self-management phase, there is a clear overlap between the TDF domains, social influences, and environmental context and resources. This is unsurprising given the interconnected nature of home, work, and social or family lives in the day-to-day lives of many people. Leveraging technology to provide increased opportunities for family involvement has clear potential and has been widely explored in other areas of health-focused research. Maintaining a balance between people’s desire to be self-reliant and their desire to be connected is also critical in designing such technology.

#### Monitoring

Many participants described becoming more aware of their body, the link between their mind and body, and *listened to their body* more after cardiac events. As described above, rehabilitation classes provided a safe zone. Monitoring was a key part of this, with close overall monitoring by health professionals and regular pulse and blood pressure monitoring. During the self-management phase, self-monitoring in daily life was common and again gave many patients confidence to continue with physical activities and push themselves. The most commonly monitored measurements reported by participants were heart rate, blood pressure, steps, sleep, and medication. Among these steps was the most frequently monitored unit. Fitbit (FitBit Inc) is the most widely used and well-known monitoring technology among participants. All patients who owned a Fitbit started using it after hospitalization. This was mainly for self-motivation and safety, and to obtain other useful insights about their body. Monitoring was also done to share information with the general practitioners,

I probably wouldn’t push myself to do things, whereas now, with the Fitbit, I try where possible to be able to fulfil my steps every day.

P18

TDF describes the behavioral regulation domain as anything aimed at managing or changing objectively observed or measured actions. Self-monitoring is an important component of this domain. This quote shows how some participants used monitoring technologies for behavioral regulation during the self-management phase. Monitoring also helped to provide ongoing insight and more personalized knowledge about their own body. However, continuous monitoring could also cause stress, and some patients liked monitoring only when they were performing physical activity. Warnings were seen as valuable, but only where something specific and unusual was detected, and not in a more routine or general way that highlights limitations:

That could actually cause more of a kind of worrying aspect to people, it could lead to more stress, having to do that and to also find if their heart rate wasn’t good, it would be more of a worry to them.

P19

It would be useful if...it could issue a warning if something irregular began to happen.

P13

This perspective suggests that for some people, long-term monitoring will work best when it is structured or compartmentalized. By combining this approach with warnings that are largely focused on irregular events, it may be possible to develop systems that provide a safe zoning effect similar to that identified in face-to-face classes in the rehabilitation phase. To achieve this monitored safe zone, it is critical that people trust the privacy of monitoring technologies. Some participants questioned the integrity and transparency of technologies and were unsure if web-based resources could be trusted. Surprisingly, others also questioned their own potential honesty when entering their own information to seek help through digital apps:

You can put into a computer whatever you like. You can say I’m a 6-foot leggy blonde, how do you advise me to get better, but you can type anything in. You’re not going to have to be honest into a computer but face-to-face...

P2

#### Capability

One of the most interesting recommendations made by the participants was that technology should act as an empowering agent. In particular, it should focus on what can be done, rather than identifying or tracking limitations. Patients believed that technology should guide them by allowing them to see what kind and how much exercise they could perform. In this way, technology would more closely mirror the guidance provided by health professionals in rehabilitation classes,

If there was any kind of technology or anything that would say to them you could actually do this after so many weeks, with care, I think so because all you get told, ‘Don’t do this’, and then you’re sitting there and you think, oh, and everything just seizes up and your confidence does go, to be honest with you.

P15

Respect for people’s autonomy was also important, with 1 participant negatively describing technology as *assertive*. To be successful, it was essential that technology respected peoples’ autonomy:

That you’re always in control of them. What they’re providing you with is information and suggestions rather than commands.

P13

It was found that patients accomplished physical activity through activities in daily life. The preferred type of physical activity for most patients was walking and gardening. Their occupation and where they lived reflected on the type of physical activity they preferred:

My husband’s a farmer. We live on a farm. We have no problem with exercise at all.

P2

As discussed above, self-monitoring is an important construct within the TDF *behavioral regulation* domain. Habit is an important construct in this domain. Alongside encouraging targeted lifestyle changes, our data suggest that long-term rehabilitation technology will be most effective if it draws on previously established healthy habits and activities of daily life. This can be combined with recommendations that emphasize capability and reinforce positive opportunities, allowing designers to build on the empowerment construct, which is emphasized in the TDF’s *Belief about Capability* domain. This overall approach is complementary to participants’ desire for a normal life and should thus be a key focus for designers.

## Discussion

### Principal Findings

**Figure 2:**
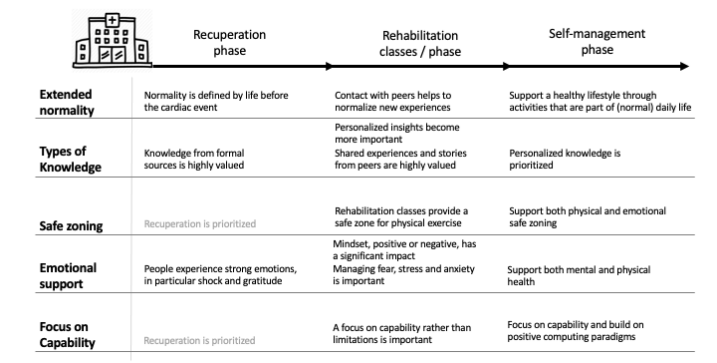
Key patient experiences and areas where technology can provide support during recuperation, rehabilitation, and self-management.

As described in the Related Work section, TDF is a synthesis of previous theories of behavioral change. Mapping the themes to the TDF domains provided us with key domains and behavioral constructs to consider in each phase after the cardiac event. The key strength of TDF is that it provides a rigorous and holistic framework for identifying a wide range of factors that impact behavior. Unsurprisingly, this has resulted in individual findings that are consistent with earlier research on health behavioral change, both in the cardiac domain and beyond. Critically, however, the use of TDF has also allowed us to see how factors that influence behavior evolve over time and identify potential sources of tension. For example, participants experienced a strong initial need for formal knowledge and access to health experts. This subsequently shifted to a desire for detailed personal insight and shared peer knowledge. We also see how participants experienced a strong desire for a normal life after surgery and how a redefinition of normality is important in long-term recovery. In this section, we discuss our findings, focusing on five key issues, namely, extended normality, safe zoning, focus on capability, different types of knowledge, and emotional support. Figure 2 provides an overview of the key points and recommendations addressed in the *Discussion*.

### Extended Normality

Existing literature has described the mundane nature of day-to-day self-care [17] and the degree to which people prefer not to be reminded of chronic health conditions [28]. We also find that a desire for normality is a strong motivating factor; indeed, it is a stated goal for many people after cardiac surgery. This creates an obvious source of tension, as lifestyle change is an important part of cardiac rehabilitation and is critical to long-term health. Given participants’ strength of feelings, it is unlikely that behavioral change strategies that run counter to the goal of normality will be successful. Interestingly, our findings show how some participants’ conceptions of normality evolved over time and suggest ways to address this challenge. We call this *extended normality*.

During the recuperation phase, normality is defined as a return to the life participants lived before their acute cardiac event. Official knowledge sources and contact with experts provided information on the recommended changes. However, in rehabilitation classes, participants also began to normalize their new experiences through social interaction and by sharing experiences with other cardiac patients. In the self-management phase, the participants who were most successful in sustaining healthy behavior were those who integrated their health management with their preferred activities of daily life, such as walking or gardening. This helped them reclaim a sense of their old routine, independence, and *normal life*. Viewed through TDF, this also engages with the importance of self-identification in either hindering or supporting healthy behavior. The study of stroke survivors by Ploderer et al [15] also highlighted the people’s efforts to manage the illness as well as everyday life activities and to reconstruct their identities.

This has led to several recommendations for technology. Critically, technologies should recognize that exceptional goals and external incentives may not be necessary. Normal life is a goal and incentive in and of itself. Care should also be taken to resolve the potential conflict that might arise between participants’ goal for normality and the lifestyle change goals recommended by professionals for rehabilitation and long-term health management. Personalized rehabilitation programs that respect personal autonomy and provide tailored recommendations linked to daily life can help address this tension. As people transition to life after surgery, technology that supports enhanced contact with peers and shared stories can also help develop a new sense of normality.

### Types of Knowledge

A previous study by Pollack et al [5] provided a detailed exploration of the experience of patients discharged from the hospital. They describe how people are often unprepared for a transition from the hospital and identified three important challenges for patients recovering from illness and needed to engage in successful self-management: (1) lack of support for health knowledge, (2) no opportunity to access resources, and (3) no opportunities to promote self-efficacy. We discuss self-efficacy in greater detail in the section focusing on capability below. Here, we consider knowledge and access. Our findings again show that people’s knowledge needs changed over time.

During the recuperation phase, people place a high value on formal knowledge, by which we mean information provided by health professionals and official sources. Much of this was standardized information about cardiac rehabilitation and lifestyle management, including standardized official booklets. Participants also sought web-based information but were often mistrusting of such sources. During the rehabilitation phase, a change occurred in the information that participants valued. Formal knowledge remained important, but participants no longer wanted generic information. They placed a high value on both shared experiential knowledge and detailed personal insight. Shared experience was facilitated through contact with peers in rehabilitation classes and occasionally through web-based support groups. As noted above, it played an important role in normalizing people’s new experiences. Personal knowledge was initially facilitated through the tailored support provided by health professionals in classes and later, although typically to a lesser degree, through self-monitoring technology.

Our findings regarding types of knowledge are consistent with a recent systematic review of barriers and facilitators of technology for cardiac rehabilitation and self-management. It also emphasized the need for technology designers to support background knowledge as well as personal and in-the-moment knowledge, where background knowledge is awareness of their medical conditions, medication, posthospital care measures, and available support systems; and in-the-moment knowledge is awareness of current body condition and changes in their body [11].

Moving forward, technologies that support different types of knowledge have significant potential. However, it was striking that many of our participants expressed the view that technology is a *solitary* thing. Within the HCI field, significant research has been conducted on the design of technologies that support social connectedness in health [51], and in personal and lived informatics [52] and the use of technology to support informal caregiving [53]. We do not have the scope to elaborate on this work at this point, except to state that the development of effective social networks in self-monitoring technologies in the health domain is clearly not a trivial task. However, research in the cardiac space will benefit from building on this earlier work.

### Safe Zoning

During the rehabilitation phase, participants liked the controlled environment, intensive monitoring, and detailed personalized support provided by health professionals. It provided insight about their current health status and increased confidence by assuring them that they were within a safe zone of physical activity. This *safe zoning* helped participants overcome emotions such as fear. Critically, it did not provide safety by reducing the activity. Rather, it provided a space where people could push themselves without the fear of overburdening their body.

Technology that supports this safe zoning on an ongoing basis is likely to be highly valuable. Importantly, safe zoning should consider not only physical, but also emotional safe zones. During the self-management phase, self-monitoring gave some patients confidence to continue physical activities and push themselves. However, many patients also did not want to be monitored continuously, as this could cause anxiety and interfere with their desire for normality. This finding is consistent with previous findings of Maitland et al [28] that cardiovascular patients were reluctant to accept unnecessary monitoring. Warnings were also considered valuable only when something unusual was detected and not in a more routine or general way. A structured or compartmentalized monitoring approach with warnings largely focused on irregular events may help to provide a *safe zone* effect similar to face-to-face rehabilitation classes. Transparency and trust in the privacy of monitoring technologies are critical for achieving this goal.

### Emotional Support

Acute cardiac events affect people both physically and mentally. In recent decades, health research has increasingly recognized and addressed the interrelated nature of physical and mental health. For example, the recognition of psycho-oncology is a key element in rehabilitation for cancer survivors [54,55].

As participants transitioned from recuperation to the rehabilitation phase, their emotions transitioned from shock and gratitude to long-term emotions. Multiple emotions built up and left unchecked can affect a person’s mental health, inducing fear, anxiety, negativity, and loss of confidence. Many patients have stressed the importance of emotional support. Family and close friends are often vital sources of emotional support. Participants pointed out that although a lot was done to educate and motivate them on physical exercise and diet, less attention was given to emotional strength. Although in-person emotional or mental support is preferred, there is increasing evidence in recent years that technology can play a significant role in providing support for mental health [56]. Examples range from systems specifically designed to integrate with traditional care [57] to the more exploratory use of voice interfaces and chatbots using artificial intelligence to provide emotional support [58]. Importantly, alongside negative emotional experiences, participants also expressed positive emotions such as gratitude and renewed appreciation of the natural world. Many also described the beneficial impact of a positive mindset and an increased sense of the link between mind and body, including an appreciation of the stress reduction in rehabilitation classes. This suggests significant potential in the application of positive computing approaches [59] that emphasize human potential and reinforce emotions such as kindness and gratitude. Approaches such as computer-supported mindfulness also have significant potential to support stress reduction and enhance the sense of a positive mind-body link [60].

### Focus on Capability

Building on the value of positive computing approaches, this study strongly suggests that designers should focus on capability rather than limitations. Particularly in the self-management phase, our participants expressed a strong desire for technology that could recognize renewed strength and make positive recommendations. They wanted technology to show what is possible by tailoring to their capabilities rather than focusing on limitations. They also wanted technology that respected their autonomy, placed them in control, and offered suggestions rather than being directive. Interestingly, some participants placed a significant value on self-sufficiency. They did not like to be helped by their families or friends. It is possible that people in this group would also consider technology as encroaching on their preference for self-sufficiency. However, we consider it more likely that autonomy-respecting and capability-focused systems will have a significant potential with this group. This analysis resonates with the conclusions of Andersen et al [20], where reintroducing patients as active diagnostic agents in the telemonitoring system showed patient willingness to take on the added workload and become actively engaged in their monitoring and diagnosis.

Through the growing capabilities of recommendation system techniques, we envision technology to be key in enabling personalized rehabilitation and self-care by focusing on individual capabilities. Tailoring recommendations for daily activities will be important in achieving this. Apps should also take into account the effect of progress awareness, wherein tailored programs based on step-by-step progress and presentation of the progress would contribute toward motivation. Previous HCI literature on person-centered recommender systems by researchers such as Konrad et al [61] and Hollis et al [62] offers valuable guidance in this area.

### Limitations

Although we interviewed a relatively diverse group of people with cardiac problems, including people who both withdrew from and attended a full rehabilitation program, it will be beneficial if future studies include more people aged less than 55 years and more people from urban areas. Although our findings are directed toward supporting patients, we understand that the opinions of caregivers are crucial and involving them will provide a broader view of the impact technology in support rehabilitation and self-care. Similarly, including health care professionals in the design process will also be crucial to the development of technologies that are acceptable and effective in improving the rehabilitation and self-management practices of patients. This work is beyond the scope of this study. Our future studies will involve both patients and health professionals and will apply co-design methods to implement systems that operationalize and evaluate the recommendations provided in this paper.

### Future Work

The *Discussion* section has identified a number of important avenues for research on the design of technology to support cardiovascular rehabilitation and self-management. Continuing to address the theoretical basis for this research will be a key focus of our work. As described in the related work section, TDF is an integrated theoretical framework composed of domains synthesized from theories and theoretical constructs relevant to behavioral change. Building on TDF, researchers in behavior change have also developed the behavior change wheel (BCW) [43,44]. This supports intervention designers in selecting intervention and behavioral change techniques by mapping the TDF domains to the BCW. BCW is based on three components, namely, capability, opportunity, and motivation (the COM-B model). It presents human behavior (B) as resulting from the interaction between physical and psychological capabilities (C), opportunities provided by the physical and social environment (O), and reflective and automatic motivation (M) [63,64]. For example, TDF domains linked to capability (C) are knowledge, skills, memory, and behavioral regulation. BCW proposes the following interventions to address factors related to capability: education, training, and enablement. In this way, BCW proposes interventions and policies for each of the three components. Building on the identification of important TDF domains and constructs in this study, application of BCW is a key priority in our future research.

### Conclusions

This paper has applied the TDF to explore the experiences of people with CVD, focusing specifically on recuperation, rehabilitation, and self-management phases after an acute cardiac event. Through these three phases, we have described how factors such as desire for normality, types of knowledge, safe zoning, connectedness, and capability impact patients. We then highlight the TDF domains that are linked to the factors arising in the three phases. Building on our findings, we have provided implications of these factors and the TDF domains in the design of technology-mediated cardiac rehabilitation and self-management.

## Appendix

Multimedia Appendix 1 Demographic information about the participants.

Multimedia Appendix 2 Interview guide.

## Abbreviations

BCW: behavior change wheel
CVD: cardiovascular disease
HCI: human-computer interaction
NHS: National Health Service
TDF: Theoretical Domains Framework
