# Supplementary material for: Transitions in Technology-Mediated Cardiac Rehabilitation and Self-management: Qualitative Study Using the Theoretical Domains Framework
Source: JMIR Cardio. 2021 Oct 14;5(2):e30428. doi: 10.2196/30428 (PMC8554673; doi:10.2196/30428)
Supplement: Multimedia Appendix 1 [file cardio_v5i2e30428_app1.docx]

Demographic information about participants.

| **ID** | **Age** | **Gender** | **Rehab class attendance** | **Main form of exercise** | **Technology used in day-to-day life** | **First cardiac incident** |
| --- | --- | --- | --- | --- | --- | --- |
| P01 | 66 | F | All | Walking | Mobile phone | 4 yrs ago |
| P02 | 70 | F | Some | Gardening, walking, looking after grandchildren | iPad, computer | 3 yrs |
| P03 | 81 | F | All | Walking | Fitbit, iPad, computer | 10 yrs |
| P04 | 65 | M | Some | Walking, exercise bike | Fitbit, iPad, computer | 3 yrs |
| P05 | 75 | F | All | Walking, gardening | Tablet | 4 yrs |
| P06 | 70 | F | All | Walking, exercise bike | Smartwatch, computer | 3 yrs |
| P07 | 83 | F | All | Walking | Mobile phone | 4 yrs |
| P08 | 58 | M | Some | Walking | Mobile phone | 3 yrs |
| P09 | 86 | F | All | Walking, gardening | No technology | 3 yrs |
| P10 | 77 | M | Some | Fishing, gardening | Computer, mobile phone | 20 yrs |
| P11 | 79 | F | All | Walking | Computer, Mobile phone | 3 yrs |
| P12 | 57 | M | All | Walking, Gym | Computer, Mobile phone | 2 yrs |
| P13 | 71 | M | All | Walking, gardening | Fitbit, Mobile phone | 4 yrs |
| P14 | 67 | F | Some | Walking, gardening | Fitbit, Mobile phone, computer | 6 yrs |
| P15 | 68 | F | All | Aqua fit, Pilates, walking | Mobile phone | 3 yrs |
| P16 | 70 | M | All | Fishing, walking | Mobile phone, computer | 7 yrs |
| P17 | 50 | M | All | Cycling, Gym | Fitbit, mobile phone, computer | 3 yrs |
| P18 | 67 | M | Some | Gym | Fitbit, computer, mobile phone | 9 yrs |
| P19 | 66 | F | Some | Walking | iPad, mobile phone | 2 yrs |
