# Supplementary material for: Transitions in Technology-Mediated Cardiac Rehabilitation and Self-management: Qualitative Study Using the Theoretical Domains Framework
Source: JMIR Cardio. 2021 Oct 14;5(2):e30428. doi: 10.2196/30428 (PMC8554673; doi:10.2196/30428)
Supplement: Multimedia Appendix 2 [file cardio_v5i2e30428_app2.docx]

## Participant Questionnaire Guide

Checklist:

1. Participant has already received the Information leaflet and has given consent.
2. Send interview date and time confirmation email to participants
3. During the interview:
   1. Introduce self and brief about the study:
   2. Start questionnaire

| **Example questions about knowledge and skills** |
| --- |
| 1. When did you first become **aware** of your cardiac condition?   (First cardiac incident, tell me what happened)   1. How **much did you know about your condition**? What **sources of information** did you use to learn about it and the impact it could have on your day-to-day life?   (Did you receive any advice or information during your hospital admission?)   1. How much did you **know about cardiac rehabilitation** and management and the steps to be taken towards it? (how did you learn) 2. Did you have to consider any **changes** in **your day-to-day life**? 3. Were there any **new skills or techniques** you had to acquire to manage your condition (for recovery/ to maintain recovery)? What are they? 4. Were you initially **confident** in your ability to make lifestyle changes after you learnt about your cardiac condition (or confident to begin the recovery process and begin day-to-day life)? 5. **Do you use technology** in your day-to-day life? If yes, what kind of technology do you use? How? (to manage cardiac condition/ health?) 6. Are you **aware of any applications/ technologies** used for cardiac management (or used to maintain health)? (e.g. Blood pressure monitor, heart rate monitor, weighing scales…) |

| **Example questions about individual goals and intentions** |
| --- |
| 1. Choice of question depends on participant’s response in previous questions.    1. If you have attended a **rehabilitation program**, how long did the program last? Tell me about it. (Did you use/ were you advised to use any technology during the rehabilitation program? Is there anything you would recommend adding to the classes/ program?)    2. If you are **using a technology** for health management, how do you use it? If stopped, why did you stop? how strong is your intention to use it for long term? Why?    3. If you are **not using a technology** for management, would you be willing to use it if it gave useful information and guidance on health? Why?   (what are the barriers in using technology?)  (If you could recommend technology to someone with a cardiac condition to help with the rehabilitation progress, what features do you think it should have?)   1. When you realised about your health condition, did the **goals** in your life change? How? 2. Are the techniques that you mentioned earlier help you **stick to your goals**? 3. Do you keep **track** of your **overall progress** towards a healthy lifestyle? How and how often? 4. Do you **remember and use** all the **information** received during the rehabilitation classes? 5. Do you feel that rehabilitation programs and/or technology can help you get the right information in order to focus on your wellbeing? (If no, what approach would you consider? or Are you satisfied with your approach towards recovery?) |
| **Questions about social and environment (sources of influence and motivation)** |
| 1. Does your **day-to-day environment impact** your ability to maintain your cardiac condition? (e.g. home, work, social environment)    1. If work environment interferes with cardiac management? How?    2. If home environment interferes with cardiac management? How?    3. If social environment interferes with cardiac management? How? 2. Does meeting with health professionals help or hinder you towards cardiac management (maintaining recovery)?   (e.g. cardiologists, consultants, nurses, physiotherapists...)   - 1. How ‘x’ impact you?  1. Do the people in your life help or hinder you towards cardiac management?   (e.g. family, friends, online communities, support groups...)   - 1. How ‘x’ impact you?  1. Do any of these: competitiveness, recognition, achievement, bragging; in any form, influence you in maintaining healthy lifestyle? 2. If you use technology, what aspects of ‘technology x’ do you like and what do you not like? (e.g. features, design...) |
| **Questions about emotional influence** |
| 1. How **did it feel** when you first realised about the cardiac condition? 2. Did your feelings **change over time**? How? 3. What was your **overall experience** of the rehabilitation process (or recovery)? 4. Were there **any major high or low points** during the rehabilitation (or recovery)?   Is there anything else you would like to add about anything we have discussed? |

Conclude: Thank you again for your time.

Ask permission to follow up later if needed.

#### **Theoretical domains framework for use in behavior change research**

1. **Knowledge**: An awareness of the existence of something
2. **Skills**: An ability or proficiency acquired through practice
3. **Beliefs about capabilities**: Acceptance of the truth, reality or validity about an ability, talent, or facility that a person can put to constructive use
4. **Optimism**: The confidence that things will happen for the best or that desired goals will be attained
5. **Beliefs about consequences**: Acceptance of the truth, reality, or validity about outcomes of a behaviour in a given situation
6. **Reinforcement**: Increasing the probability of a response by arranging a dependent relationship, or contingency, between the response and a given stimulus
7. **Intentions**: A conscious decision to perform a behaviour or a resolve to act in a certain way
8. **Goals**: Mental representations of outcomes or end states that an individual wants to achieve
9. **Memory, attention and decision processes**: The ability to retain information, focus selectively of aspects of the environment and choose between two or more alternatives
10. **Behavioural regulation**: Anything aimed at managing or changing objectivity observed or measured actions
11. **Environmental context and resources**: Any circumstance of a person’s situation or environment that discourages or encourages the development of skills and abilities, independence, social competence, and adaptive behaviour
12. **Social/Professional role and identity**: A coherent set of behaviours and displayed personal qualities of an individual in a social or work setting
13. **Social influences**: Those interpersonal processes that can cause individuals to change their thoughts, feelings, or behaviours
14. **Emotion**: A complex reaction pattern, involving experiential, behavioural, and physiological elements, by which the individual attempts to deal with a personally significant matter or event
